# Using *Trellis* software to enhance high-quality large-scale network data collection in the field

**DOI:** 10.1016/j.socnet.2021.02.007

**Published:** 2021-07

**Authors:** Alina Lungeanu, Mark McKnight, Rennie Negron, Wolfgang Munar, Nicholas A. Christakis, Noshir S. Contractor

**Affiliations:** a Northwestern University, 2240 Campus Drive, Evanston, IL 60208, United States; b Yale University, 17 Hillhouse Avenue, New Haven, CT 06511, United States; c George Washington University, 950 New Hampshire Ave, Washington, DC, 20052, United States

**Keywords:** Mobile social network survey technologies, Rural network data collection, Software data collection, Graphical interface, Online surveys

## Abstract

*Trellis* is a mobile platform created by the Human Nature Lab at the Yale Institute for Network Science to collect high-quality, location-aware, off-line/online, multi-lingual, multi-relational social network and behavior data in hard-to-reach communities. Respondents use *Trellis* to identify their social contacts by name and photograph, a procedure especially useful in low-literacy populations or in contexts where names may be similar or confusing. We use social network data collected from 1,969 adult respondents in two villages in Kenya to demonstrate *Trellis*’ ability to provide unprecedented metadata to monitor and report on the data collection process including artifactual variability based on surveyors, time of day, or location.

## Highlights

• *Trellis* network data collection platform enables name disambiguation.
• *Trellis* network data collection platform enables multiple language interviews.
• *Trellis* report allows for real-time monitoring of data collection.
• *Trellis* network data collection platform can be used offline in remote areas.

## Introduction

People’s knowledge, attitudes, and behaviors depend on the knowledge, attitudes, and behaviors of the people to whom they are directly or even indirectly connected in their face-to-face social networks. Social phenomena diffuse across network ties, and this diffusion process is crucially relevant in many situations, and in particular with respect to public health.

For instance, studies examining behavioral barriers to adoption of modern contraception have shown that people’s adoption decisions are not made in a social vacuum; rather, information about potential side-effects, costs, and benefits associated with various family planning methods travels through interpersonal channels (Behrman et al., 2002; Casterline, 2001; Kohler et al., 2001; Montgomery and Casterline, 1996). Demographic and health surveys conducted in 51 low-income countries have shown that, while the availability of contraceptives has markedly improved, behavioral barriers have become the main reason for non-use. In particular, women in low-income countries report that the main obstacles to the adoption of modern contraception include perceived side effects and opposition by significant others (Sedgh and Hussain, 2014).

Interventions aimed at increasing the speed and scale of modern contraception adoption typically treat behavior change separate from the social contexts in which such changes occur. This approach leads to omissions in the design of behavior change interventions, such as: (i) unclear or insufficient knowledge about how behaviors take shape, are maintained, or are changed (Glass and McAtee, 2006), (ii) lack of understanding about the effects of social and collective norms on individual, group, and community-level behavioral trends (Bond et al., 1999) and, (iii) theorizing that omits the social and cultural dynamics through which social networks can moderate the diffusion of reproductive health knowledge, attitudes, and practices (Gesell et al., 2013). Overcoming these research gaps requires refocusing on the social and cultural dynamics that drive adoption barriers such as opposition to use and perceptions of health risks, particularly at the district and community levels.

Social network theories, methods, and tools have the potential to substantially address the “blind spots” identified above. Most behavior change theories acknowledge the roles that social networks and social norms play in influencing human behavior and individual, group, and collective health (Valente, 2010). Social network researchers have developed data collection methods and modeling tools that provide valuable insights into the ways in which behaviors diffuse through the imitation of influential others, including in less-industrialized settings (Perkins et al., 2015). For example, Alvergne et al. (2011) collected friendship network data in four villages in southern Ethiopia to investigate the effect of social networks on the spread of modern contraception. Onnela et al. (2016) used paper-based questionnaires to collect information about network contacts to investigate the polio vaccine hesitancy in India. Kamya et al. (2017) conducted structured social network surveys with key individuals involved in the HPV vaccine application process in Uganda. Banerjee et al. (2013) collected sociocentric data on a small population in 75 villages in rural Karnataka, in Southern India; while the authors collected household characteristics data from all in the villages, individual demographic and network data were collected from only half of eligible households (Shakya et al., 2015). Finally, Apicella et al. (2012) used paper photographs printed on posters taken to the field to ascertain the social networks of 205 Hadza foragers in Tanzania. These examples show that while social networks are important in global health practice, the collection of such data is a difficult process.

The current study aims at demonstrating how a recently developed mobile social network data collection platform (i.e., *Trellis*) can overcome many of these limitations. By doing so, *Trellis* helps unleash the potential of social network theories and methods to significantly advance our ability to understand the adoption of family planning techniques in remote rural contexts specifically, and societal practices in large, underserved, and hard to reach populations, more generally.

### Social network data collection platforms

Social network researchers have begun to explore how the Web and hardware devices such as touchscreen tablets and mobile phones can be leveraged for improving the efficiency and effectiveness of collecting social network data from respondents. For instance, graphical data collection software can help respondents directly answer questions about their network contacts, which in turn reduces data collection difficulty and error that typically occur with more indirect methods (Coromina and Coenders, 2006; Matzat and Snijders, 2010; Stark and Krosnick, 2017; Tubaro et al., 2014; Vehovar et al., 2008). For example, IKNOW (Contractor et al., 1998), as well as its later C-IKNOW version (Huang et al., 2008), were among the first web-based software tools designed for social network data collection, analysis, and investigation (Huisman and Van Duijn, 2005). These tools were designed around real-world problems, such as matchmaking potential collaborators, and they could store and analyze both egocentric and sociocentric network data. C-IKNOW’s visualization suite enabled dynamically generating visualizations of any sub-network without data preparation, making it easy for users to get different networks and layouts by clicking a single button. One example of the IKNOW application was the collection of social network data among a sample of organizations coordinating services for the Mexican immigrant community in Chicago (Wali et al., 2006). A more recent tool, GENSI (Stark and Krosnick, 2017), allows respondents to describe all network contacts and their characteristics all at once via a graphical representation of their networks. Network Canvas (Hogan et al., 2019, 2016) provides an intuitive interface for respondents to share information about their egocentric networks while interacting with a visual representation of their network on a touch-screen device. This platform is particularly convenient for respondents to provide data about sensitive social network relations (such as male sex with male) without having to verbalize them for the interviewer.

Other software packages such as VennMaker (Gamper et al., 2012), EgoWeb 2.0 (Kennedy et al., 2017), and EgoNet (McCarty et al., 2007) similarly provide an immediate visualization of the participant’s network during the interview process. For example, Tucker et al. (2009) used EgoNet tool to collect egocentric network data on homeless women residing in temporary shelters in Los Angeles County. These tools have been particularly useful among literate respondents who have the ability – and in some cases appreciate the privacy – to self-report their network ties.

While there is a growing number of digitally powered network data collection platforms, they differ along some key dimensions. Table 1 provides a comparison of network data collection digital platforms along 10 dimensions: (1) Some are focused more on egocentric while others focus more on sociocentric network data. (2) Some allow the collection of data offline (when not connected to the internet), while others require live connection to the internet. (3) Some are geared for respondents to directly provide their responses, while others are designed to assist data collection by a surveyor. (4) Some are web-based while others are developed as apps and yet others offer both. (5) Some have a graphical interface while others use a text/roster interface. (6) Some have the capability to capture GPS location of the respondent while others don’t. (7) Some have the capability to capture associate photographs with respondents while others don’t. (8) Some offer functionality only in English, others in pre-selected multiple languages, and yet others allow switching between multiple languages within a single survey administration. (9) Some allow data collection and network graphical visualization while others don’t. Finally, (10) most allow monitoring of statistics about data collection (completion rates, duration of time spent on each survey, comparisons of these measures based on characteristics of the surveyor and respondent), but not via a real-time dashboard. As Table 1 indicates, the platform we discuss here, *Trellis*, is often one of a few (or in some cases the only one) offering certain features that make it specifically suitable for assisting surveyors (not respondents directly) to collect sociocentric network data from a large population of potentially hard-to-reach (geographically and electronically) respondents who vary in their levels of literacy.

**Table 1:** Network Data Collection Tools: Features.

	Egocentric vs sociocentric	Online vs offline data collection	Surveyor-administered vs self-administered	Web-based vs app-based	Graphical interface vs text/roster interface	GPS capability
Trellis	Sociocentric	Online & offline	Surveyor-administered	App-based	Text/roster	Overlay GPS coordinates on the map
IKNOW	Sociocentric	Online only	Surveyor-administered	Web-based	Text/roster	Not available
CI-KNOW*	Sociocentric	Online only	Surveyor-administered	Web-based	Text/roster	Not available
Network Canvas	Egocentric	Online only	Self-administered	Web-based & App-based	Graphical	Not available
GenSI	Egocentric	Online only	Self-administered	Web-based	Graphical	Not available
VennMaker	Egocentric	Online & offline	Surveyor-administered	Web-based	Graphical	Not available
EgoNet*	Egocentric	Online & offline	Surveyor-administered	Web-based	Graphical	Not available
EgoWeb 2.0	Egocentric	Online & offline	Surveyor-administered	Web-based	Graphical	Not available

	Photographic census	One vs multiple languages	Network data visualization and analysis	Data collection monitoring	Website
Trellis	Yes	Switch between multiple languages during survey administration	Collection	Real-time dashboard	https://trellis.yale.edu/
IKNOW	No	English only	Collection & Visualization & Analysis	Pre-processing followed by monitoring	https://web.archive.org/web/20061215231248/http://www.spcomm.uiuc.edu/teclab/iknow/
CI-KNOW*	No	English only	Collection & Visualization & Analysis	Pre-processing followed by monitoring	http://sonic.northwestern.edu/software/software-archive/c-iknow/
Network Canvas	No	English only	Collection & Visualization	Pre-processing followed by monitoring	https://networkcanvas.com/
GenSI	No	Multiple languages	Collection & Visualization	Pre-processing followed by monitoring	http://www.tobiasstark.nl/GENSI/GENSI.htm
VennMaker	No	Multiple languages	Collection & Visualization	Pre-processing followed by monitoring	https://www.vennmaker.com/?lang=en
EgoNet*	No	Multiple languages	Collection & Visualization	Pre-processing followed by monitoring	https://sourceforge.net/projects/egonet/
EgoWeb 2.0	No	Multiple languages	Collection & Visualization	Pre-processing followed by monitoring	https://www.qualintitative.com/wiki/doku.php/start
					

* archived.

## Trellis Survey Tool: Mobile social network data collection platform

### Description

The *Trellis* survey tool^1^, developed by the Human Nature Lab at Yale Institute for Network Science, has been designed to collect high-quality multi-relational sociocentric network and behavior data. *Trellis* consists of an Android app to collect field data, a web application for survey design, and data storage. The *Trellis* web app also facilitates the mapping of residences using satellite imagery. The Android devices can be used offline and later synced with a central Web server. An especially valuable feature of *Trellis* is that the respondents can identify their social contacts not only by name but also by their photographs. This allows for accurate mapping of social networks even in low-literacy populations or where names may be similar or confusing.

### History

Development of the software that would eventually become *Trellis v1* was started in 2014 by one team of consultants (Alcanzar Software Solutions) and completed in 2015 by a second team (Pixel and Line / Vynyl). *Trellis v1* was used to map networks and collect data from 32 villages in Honduras (Kim et al., 2015) and for collecting network data from 176 villages in Honduras (Shakya et al., 2017b). The research team in charge of collecting data from Honduras faced two challenges. The first challenge was accurately mapping the social networks without using name entry or lookup alone. In a low-literacy population, it is very difficult for respondents to uniquely identify people they nominate by name alone as there may be name collisions, names may vary in spelling, or respondents may identify people by their nicknames. The second challenge was that the area the research team would be working in had very limited access to wireless data; as a result, responses would have to be collected offline and later synchronized to a central server.

One of the data collection tool’s requirements was that the software would be open-source. Open Data Kit (ODK) was one option evaluated that fulfilled one of the two requirements, having offline data collection and synchronization capabilities. It did not have the features needed to perform a full photographic census and to facilitate mapping the social network. The research team considered contributing a component to ODK to add this feature to the existing platform. However, because the team valued having ownership over the platform and the ability to customize the tool overall to meet the needs of the survey team, the final decision was to build a new tool from scratch based on our own social network data collection requirements.

Immediately following the completion of *Trellis v1*, a second team of developers began working on *Trellis v2* in order to correct issues and add additional features. For example, *Trellis v1* was dual-language (English / Spanish) only. *Trellis v2* included the ability to translate respondent facing text into multiple languages. *Trellis v2* also added the ability to ask follow-up questions about nominated alters (such as, "Who do you go to for health advice?” Margot, Jean. “How many hours a week do you spend, on average, with Margot?” “How many hours a week do you spend, on average, with Jean?”). *Trellis v2* was used to collect data in the study reported here.

In 2018, the Human Nature Lab team began work on *Trellis v3*. *Trellis v3* is a redesign of the application using the Apache Cordova framework for mobile applications and was released as open-source software in 2019. The advantage of using Trellis is that it allows the development team to use the same application code in the web app as in the tablet, and allows surveyors to conduct interviews on the server directly (e.g., phone interviews or follow-up questions). The Apache Cordova framework also allows Trellis to be ported to iOS in the future.

In addition, compared to paper survey instruments, *Trellis v3* stores an action log for every interview which allows administrators to analyze their surveyors’ interactions with the tool and even correct, post-facto errors in the survey instrument while preserving the surveyor’s input. Finally, the synchronization system is efficient when working with large uploads and downloads and to store a log of changes made by each device as it is synchronized with the server. More recently, *Trellis v3* has received several security updates including the ability to create different roles, restrict their access to the application, and assign roles to users.

When compared to paper survey instruments, *Trellis* has many other advantages. These include avoiding the time and possibility of errors for manual data-entry, automatically asking or skipping questions based on previous responses, and customizing question text based on previous responses (fills). *Trellis* has been designed for surveyor-administered surveys and supports surveys either in-person or over the phone.

While, as a sophisticated client-server application, *Trellis* provides many benefits, it also requires a technical team to set-up and manage the infrastructure which may increase the cost of surveys (though minimizing the time). *Trellis* does not currently exist as a Software as a Service (SaaS) application and a team that uses *Trellis* should be prepared to set up and maintain their own Trellis server. Finally, at this time, *Trellis* has only been tested and used in the field using Android tablet devices. *Trellis v3* has the potential to be ported to iOS due to its use of the Apache Cordova framework, but this work has not yet been done.

### Technical details

**Fig. 1:**
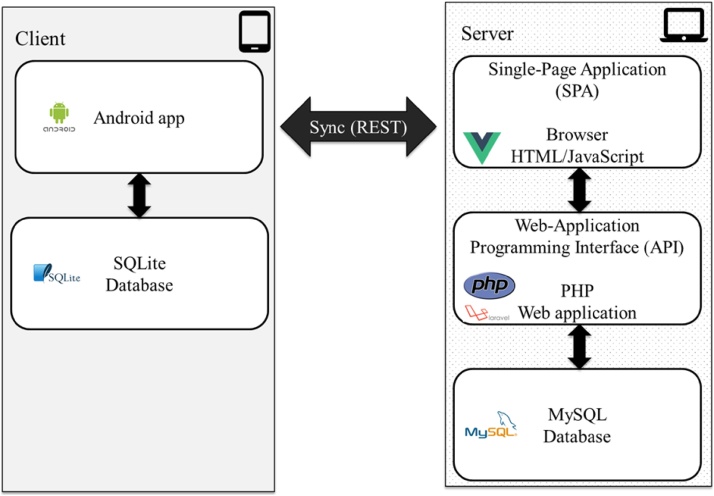
Trellis Architecture.

*Trellis* is a client-server application that consists of an Android native app on the client-side, and a single-page web application, and a web-application programming interface on the server-side. Fig. 1 illustrates the *Trellis* architecture.

The Android native app was developed in Java and runs on Android Runtime on any Android phone or tablet. This application can function as a stand-alone data collection tool and interfaces with the server through a data synchronization process (upload/download). Surveyors in the field use the Android app to add and search for respondents or locations and to conduct surveys. The Android mobile platform was chosen because devices using it are inexpensive and widely available, especially for collecting data in rural and underdeveloped regions. Additionally, the Android operating system is Open Source and has a rich ecosystem of many open-source software libraries. Limiting the mobile platform to Android only allowed for developing a native application: with buttons and menus that look the same as other apps developed for Android.

The single-page web application (or SPA) was developed in JavaScript and HTML5 using many open-source web technologies but primarily the AngularJS (angular.io) web framework and Bootstrap front-end framework. A SPA dynamically changes the current page rather than navigating to new pages on the server and appears more like the mobile apps and installed applications that users typically interact with. Study administrators use the web application to create study forms, register devices, add users, export data, view, and print study maps, and manage the synchronization process.

The server-side web-application programming interface (or API) was developed using PHP and the Lumen (lumen.laravel.com) micro-framework. The web application interfaces with the *Trellis* database via the web API and the Android app synchronizes the data with the server using the API.

### Key features

#### Ability to overlay GPS positions on to a map

*Trellis* uses the host device's location services to record the position (longitude, latitude, and altitude) of the device when a location or respondent is added, and a new interview is started. Locations added to *Trellis* may be viewed in the web application as a layer on top of OpenStreetMap^2^ map data. Study administrators may use these maps to plan out a survey team's routes and to divide a study area among surveyors.

#### Ability to conduct a photographic census of study participants

When a surveyor adds a respondent to the *Trellis* app, the software prompts the user to enter a name and take a photo of the respondent using the device's built-in digital camera. *Trellis* creates a respondent record with the name and photograph identified by a generated unique identifier (UUID version 4^3^). The respondent search feature allows a surveyor to search for a respondent using all or parts of their name and positively identify the respondent by photo. The verification of the identity of the respondent by photo both allows for surveyors to accurately re-identify respondents on future waves of data collection, and also allows respondents to identify and nominate alters even when they do not know the full name or the name is common in the study population. This allows for accurate mapping of social networks even in low-literacy populations or where names may be similar or confusing.

#### Translation of survey instruments and user interface into multiple languages

Study administrators can translate all respondent-facing text in surveys into multiple languages. *Trellis* has the capability to save and display multiple languages. A surveyor can change the display language during survey administration, which affects both question text and response options. This allows the surveyors to administer interviews in multiple languages without doing translation on the fly that may lead to differences in phrasing that might bias the respondent and adversely impact study results. Study administrators can also translate surveyor-facing text such as button labels and menu items into the surveyor's preferred language.

#### Name-generator (relationship-eliciting) question type for respondent identification of ego-alter relationships

*Trellis* is primarily a tool for survey administration. In addition to supporting standard question types (e.g. multiple choice, free text, numeric entry), *Trellis* also provides a “relationship” question type for eliciting relationships where the answer to the question can be one or more respondents from the subject pool. *Trellis'* respondent search function allows for a nominated alter to be quickly located by name or initials in combination with a photograph to verify that the correct subject has been located.

#### Offline data collection and synchronization with a central server

*Trellis* was designed to collect data in locations with limited access to wireless networks and is fully functional even when offline. The Android phone or tablet downloads all of the data it needs from the server in order to conduct data collection for the day. After downloading surveys and respondent pools from a central server, each device is capable of adding new respondents or households and conducting surveys while offline. When the device is returned to an area with network connectivity, such as the study office, the collected data is uploaded to the central server.

#### Real-time monitoring and access to survey metadata

*Trellis* offers real-time monitoring of data collection efforts via a report to assess the performance of the surveyor staff, tracking the location and the time of the survey, as well as noting any variability or biases between surveyors (such as differences in survey administration processes). This enabled agile adjustments to survey administration. *Trellis* provides valuable additional information for researchers to better report data collection processes and to identify limitations in the datasets they have gathered. These include detailed records on repeat and/or truncated visits, interview duration (for various segments of the survey), and variability in these measures across surveyors, time of day, and specific geographical location. The timestamp associated with each respondent’s recall of their social network alters also enables researchers to ask heretofore challenging research questions about the cognitive processes invoked by individuals to explain the sequence and patterns by which individuals reconstruct their network.

#### Survey study file export 4

*Trellis* allows the export of the study file in a format that can be built into a codebook. Specifically, *Trellis* offers the possibility to import / export study in the JSON format^5^ .

## Case study: Using *Trellis* to conduct sociocentric network data collection in rural Kenya

We now describe the use of the various functionalities offered by *Trellis* in the Kenyan context. Specifically, we describe how we used the various functionalities of *Trellis* to set up data collection, to monitor the progress of data collection, identify potential problems in close to real-time, and compute novel metrics to evaluate the quality of data collection based on attributes of the surveyors and the respondents.

The overarching goal of The Bill & Melinda Gates Foundation’s Family Planning program is to “bring access to high-quality contraceptive information, services, and supplies to an additional 120 million women and girls in the poorest countries by 2020 without coercion or discrimination, with the longer-term goal of universal access to voluntary family planning.^6^ ” The program seeks to identify access barriers and funding gaps to family planning, to develop and test interventions, to monitor changes in contraceptive use, and help countries track annual progress toward their goals and improve program performance. The FP2020 represents the Family Planning 2020 Performance, Monitoring, and Evaluation 2020 survey program, a global initiative that aims to increase contraceptive use in low-income countries. The Family Planning 2020 is hosted by the United Nations Foundation and includes as partners the Bill & Melinda Gates Foundation, UK’s Department for International Development, the US Agency for International Development among others.

Along with other partner organizations, the Gates Foundation’s initial focus was on the development, clinical trials, testing, and delivery of reliable and inexpensive modern contraceptive methods to rural underserved populations. However, more recently there was a growing realization at the Foundation that delivering reliable and inexpensive modern contraceptive methods was a necessary, but not sufficient, condition for their actual adoption by the population. This focus on “scaling up” the adoption of modern contraceptives prompted the Foundation to invest in research that would offer a more careful understanding of the ways in which individuals’ attitudes towards modern contraceptive methods were being shaped by their social networks. In response to the Foundation’s goal, we were charged with developing a set of systems-science measurement tools to help us gain a better understanding of the collective and individual causes for the non-use of modern contraceptive methods. Our study was designed to bridge population health concerns (enhancing the toolkit in use for measuring community-level behavior change in the family planning space) and the theories and methods from system sciences, in particular social networks. Using social network theory (Valente, 2010), we conceptualized change in modern contraceptive (MC) use as being mediated by mechanisms of social influence (Cialdini, 2009; Contractor and DeChurch, 2014) and social selection that directly contribute to the adoption of MC. The study was designed to test the applications and limitations of using a relational approach to characterizing how social network structure and community attitudes, beliefs, and norms (injunctive and descriptive) influence the use (or non-use) of modern contraception in Kilifi County, Kenya.

### Study setting

We first conducted qualitative research in three villages in Kilifi County to inform the subsequent design of the social network study component. This approach not only guided the design of the social network survey but also helped the team contextualize and conceptualize the beliefs, attitudes, and norms that guide people’s family planning behaviors. The qualitative research led to important insights and in particular the realization that in all three villages studied, the main source of perceived negative effects for non-use of MC was the belief that MC use at a young age or before childbirth can make women infertile; therefore, when women observed other women not getting pregnant after using MCs, they attributed infertility to the use of contraception. In the villages we studied, it was widely believed that wives were expected to have a child within one year of marriage, and additional children shortly after. This pressure came from both the family and from the community as a whole: people would speak negatively about couples that are not “producing” enough children quickly enough. Overall, the social consequences of infertility were devastating for women (Sedlander et al., 2018).

We selected two communities in Kilifi County on the basis of their modern contraceptive prevalence rate (MCPR) estimated by the Family Planning 2020’s Performance, Monitoring, and Accountability 2020 (PMA2020) survey program, a global initiative that aims to increase contraceptive use in low-income countries. One of the two selected communities was undergoing a relatively advanced stage of fertility transition, with an MCPR of 44.4 %, while the second represented an earlier stage of transition, with a lower MCPR of 10.3 %. While both communities were inland, the higher MCPR community was more geographically accessible than the lower MCPR community.

During our study’s implementation, we needed to make an important decision on how to address the complexity of data collection at such a scale. The challenges of collecting in-person sociometric network survey data in geographically remote villages with low-literacy populations are well known and can introduce errors if not properly addressed. To reduce these errors, we decided to use a software platform, *Trellis*, designed to collect high-quality multi-relational social network and health behavior data. *Trellis* was designed by the Human Nature Lab at Yale’s Institute for Network Science for a sister social networks project in Honduras, also funded by the Gates Foundation. *Trellis* comprises an Android application for data collection, a web application for survey design, and a data storage component. *Trellis* Web also facilitates the mapping of houses and other living structures using satellite imagery. The Android devices can be used offline and later synced with a central web server. An especially valuable feature for the research team was that respondents could identify their social contacts not only by names but also by photographs. This allowed a high degree of accuracy when mapping the social networks of two whole villages where names were similar. Since our local partners at the International Center for Reproductive Health-Kenya (ICRHK) were already using an Android platform in their PMA2020 work, the using *Trellis* on Android served to prototype sociocentric network data collection in a challenging environment. Given existing capabilities on the ICRHK PMA2020 team, we also expected that the learning demands would be manageable by both data collectors and supervisors.

A key catalyst in facilitating our entree, access, and ability to work with ICRHK was brokered by our common connections with the Gates Foundation. The Gates Foundation has invested heavily in Family Planning issues and has built strong and enduring partnerships with local government and non-government health entities in Kenya. These connections proved invaluable in helping us forge a trusted and productive partnership with ICRHK. It was also key to consider this as a partnership since we learned from ICRHK’s experience conducting surveys on sensitive health-related issues in Kenya. And, they, in turn, learned from us about the special issues when conducting social network surveys with which they had no prior experience. The partnership benefited immensely by scheduling face-to-face meetings in the US and extended field visits to Kenya by principal investigators and graduate students from the US.

The survey was conducted by a team of experienced surveyors from ICRHK. Census data collected by ICRHK in the two selected communities indicated that they had 2,890 residents that were 15 years or older. Including all of these residents allowed us to generate large-scale sociocentric networks that captured both direct and indirect ties and that provided a complete picture of the connections among all the individuals (actors) within each of these two communities.

The study protocol was reviewed and approved by the Institutional Review Boards (IRBs) of University 1 and University 2 where study authors resided at the time of data collection and by Kenyatta National Hospital/University of Nairobi’s Ethics and Research Committee (KNH-UoN-ERC).

### Study design

The design of the survey was informed by standard principles (Marsden, 1990; Monge and Contractor, 1988; Shakya et al., 2017a). Our questionnaire sought to gauge people’s perceptions and usage of family planning in order to examine the factors that influence people’s attitudes and behavior related to family planning. Participants were asked to identify their social network alters on a host of relations including, but not limited to, who they spoke with about family planning. Table 2 presents the social network relations collected and the exact wording of the survey. For each alter they identified, they were also asked to report, based on their perceptions, information about the alter including demographics, as well as attitudes about, and use of, contraceptives.

**Table 2:** Social Network Questions.

Relation	Question text in the survey
***MC and child spacing***	
Talk about MC	Who did you talk with about medical methods of family planning in the past year?
Talk about child spacing	Who did you talk with about issues such as having a child and child spacing in the past year?
Health	
Seek health advice from	Who did you seek advice from about general health-related matters (not just medical methods of family planning) in the past year?
Came for health advice	Who came to you for health advice in the past year?
***Trust***	
Talk about private issues	Who did you trust most to talk about something personal or private in the past year?
Borrow money from	Who would you feel most comfortable asking to borrow 500 shilling from if you needed it for the day?
Asked to borrow money	Who do you think would feel most comfortable asking you to borrow 500 shilling for the day?
***Social***	
Spend free time with	With whom did you spend a lot of free time in the past year?

Given the relatively unique context, we relied heavily on formative qualitative research to help inform the social network survey and better identify the potential reasons for non-use of MC (Sedlander et al., 2018). The qualitative research component included key informant interviews and focus group discussions that helped identify the boundary of the network, specific social network relations to be surveyed, and the specific phrasing for survey questions. In addition, the qualitative study informed the categories of network alters (parents, siblings, spouses, in-laws, health practitioners, religious leaders, teachers, village elders, etc.) with whom individuals discussed family planning issues. These were important because they served as credible probes by assisting individuals to identify specific alters in their network.

**Fig. 2:**
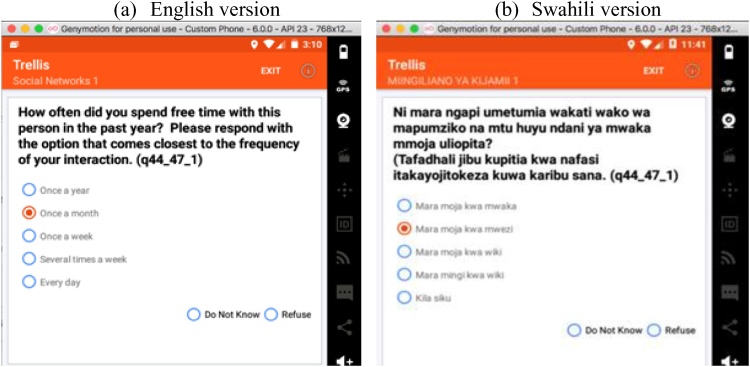
Trellis: Example of multiple language question.

The survey was translated to Swahili (and back translated to English for validation by an independent team) by a local translation firm hired by ICRHK. Both the English and Swahili versions of the survey were uploaded to the Web interface offered by *Trellis*. Fig. 2 presents an example of relationship-type question in English (Fig. 2a) and Swahili (Fig. 2b).

**Fig. 3:**
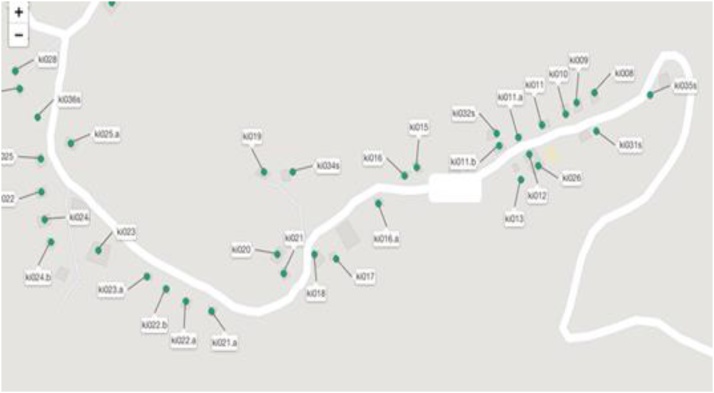
Trellis: Location function.

The surveys were conducted by a team of 11 surveyors selected and managed by ICRHK. ICRHK maintains an updated census with a numbered listing on a map of all households in communities within Kilifi county, along with names and ages of all household members. The map of all households and their GPS location was available. Fig. 3 displays a mock example of how OpenStreetMap was embedded in the *Trellis* app.

### On-site training and pilot field testing

We conducted a three-day on-site workshop to train the ICRHK’s research team and local data collectors on study design, research objectives, data collection techniques and technologies, IRB requirements, best-practices and ethical principles. While surveyors had experience with FP2020 surveys, they lacked specific experience with conducting social network surveys. Likewise, while the ICRHK had experience using mobile phones to collect survey data in households, they had no experience using mobile phones to conduct large sociocentric network surveys with large rosters that included the names and photographs of all adult community members. Therefore, we trained them on the specific challenges associated with collecting social network data using mobile technologies, such as *Trellis*. We also impressed on them the value of social network data and the empirical criticality of securing a high response rate as compared to non-network surveys.

The first day of general training on conducting social network surveys was followed by a half-day of training on the use of the *Trellis*, the mobile social network data collection platform they would use in place of a pen-and-paper survey to collect data from respondents. We trained them on two separate waves of “surveys” they would conduct in the field. The first wave, the “digital photographic census” entailed soliciting each respondent to provide, via the *Trellis* app on the mobile phone, a close-up digital portrait photograph as well as a second photograph standing in front of her/his house. These two photographs were automatically linked in *Trellis* with each unique respondent. The purpose of these photographs was to disambiguate each respondent from others who might have shared the same or similar names. This paved the way for the second wave survey, the “social network survey”, conducted at a later point in time, at which point respondents were asked to report their various network ties. Respondents were able to definitively identify alters not only by their names but, also, by reviewing their photographs and their household on the *Trellis* app.

Upon satisfactory completion of the 3-day on-site training, the team embarked on a 5-day pilot-testing of the survey in the field. In order to avoid contaminating the two test communities, we conducted the pilot field study in a third community with medium MCPR. Our partner, ICRHK, was able to provide us census data about this third community; such data were uploaded into *Trellis*. One hundred participants were randomly selected and consented by surveyors before doing the pilot survey. Participants were encouraged to raise any confusions or questions about the wording, logic, and meaning of the survey items used. The results of the pilot tests, along with the feedback gathered from the participants, helped fine-tune the instruments and troubleshoot logistic and technical issues that arose with the collection of network data via *Trellis* questionnaires. The first two days were devoted to field testing the first wave of surveys – soliciting photographs from respondents. The last three days were devoted to field testing the second wave of surveys – soliciting their social networks as well as other data. Each day of pilot testing in the field consisted of about 5 h of travel to and from the site, four and a half hours of interviewing respondents and 1 1/2 h of debriefing.

A significant challenge encountered in the field was that respondents gave few names (1∼2) when answering social networks questions and in particular the question regarding family planning methods. This ran counter to the qualitative findings about the general practices in these villages. One possible reason was that respondents were suspicious and thus reluctant to name people fearing that it might be used by government agencies against them. Some respondents expressed concerns and asked questions, while others did not ask but gave little information. Given this challenge, the surveyors were provided with two solutions: First, to explain why we are asking respondents to name their social networks, helping them understand the research objective. (e.g., “We ask this question because we want to understand how ideas and behaviors spread in this village.). The second solution was to emphasize the confidentiality of their identity and their alters’ information (e.g. “No one in this village will know that you mention this person besides our research team. We will not use this information to identify the person you name. This information will only be used for doing research.”).

Additionally, surveyors were instructed to probe tactfully by: (1) giving examples of categories of people they might mention as alters, such as partners, relatives, friends, etc.; (2) mentioning specific activities, such as fetching water, going to church, and chatting in front of your house; or (3) simply giving more time for respondents to think.

Finally, we reordered the social network questions by starting with more general questions before zooming in to specific topics. We began by asking the social network question, “With whom did you spend a lot of free time in the past year?” and then moved to general health matters, health advice and, finally, to specific family planning methods (i.e., “Who did you talk with about medical methods of family planning in the past year?”).

Following the on-site training and pilot field testing, we convened a one-day wrap-up session where we discussed each surveyor’s reflections on the training/piloting and solicited a snapshot of the surveyors’ experience. The following themes emerged at the end of the training:

● *Using Trellis was challenging at first, but things went more smoothly as they practiced more in the field;*
● *Mastering troubleshooting skills helped build confidence;*
● *Getting more familiar with the village to easily find the household;*
● *This was a great learning opportunity to learn social networks, make friends, and hone communication skills.*
● *Some surveyors mentioned that this experience will help them find jobs;*
● *Using Trellis to take pictures was fun and exciting.*

### Data collection process

**Fig. 4:**
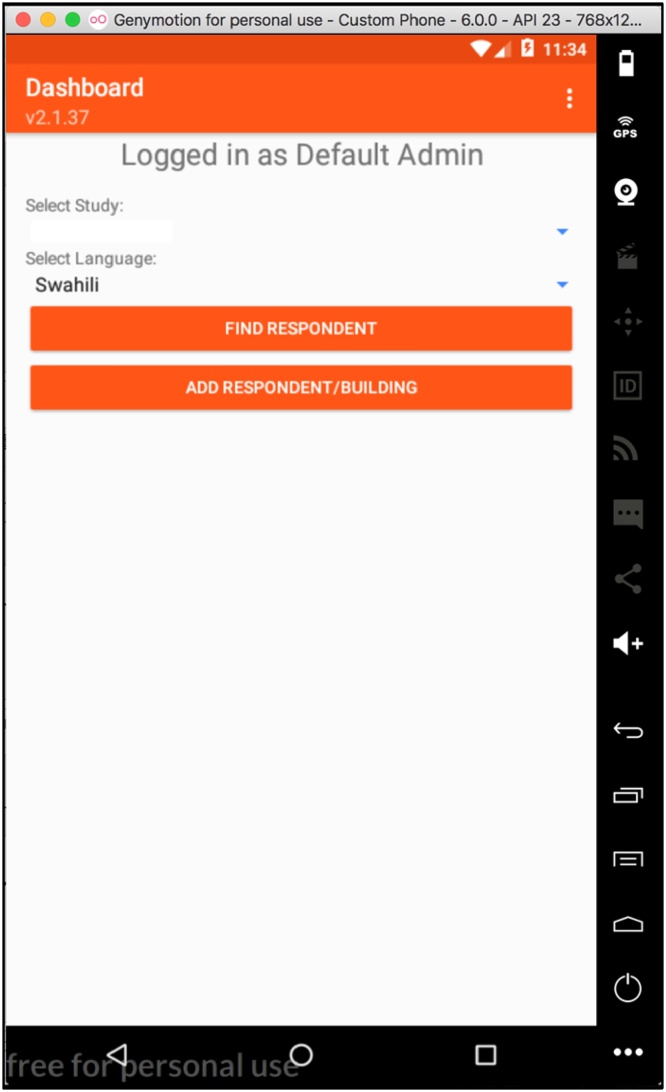
Trellis: Example of login interface.

Surveyors visited every house in the two communities to recruit participants, obtained their consent, and conduct the survey by asking questions using the *Trellis* app on smartphone in Swahili or English. Fig. 4 presents surveyors’ login page on the smartphone. The data collection was anticipated to last seven weeks but was delayed due to elections and subsequent seasonal challenges. This entailed training 6 more surveyors and offering refresher courses to the remaining ones. This process was conducted to a satisfactory conclusion by the ICRHK team. The data collection lasted approximately 3 months.

#### Wave 1: digital photographic census

**Fig. 5:**
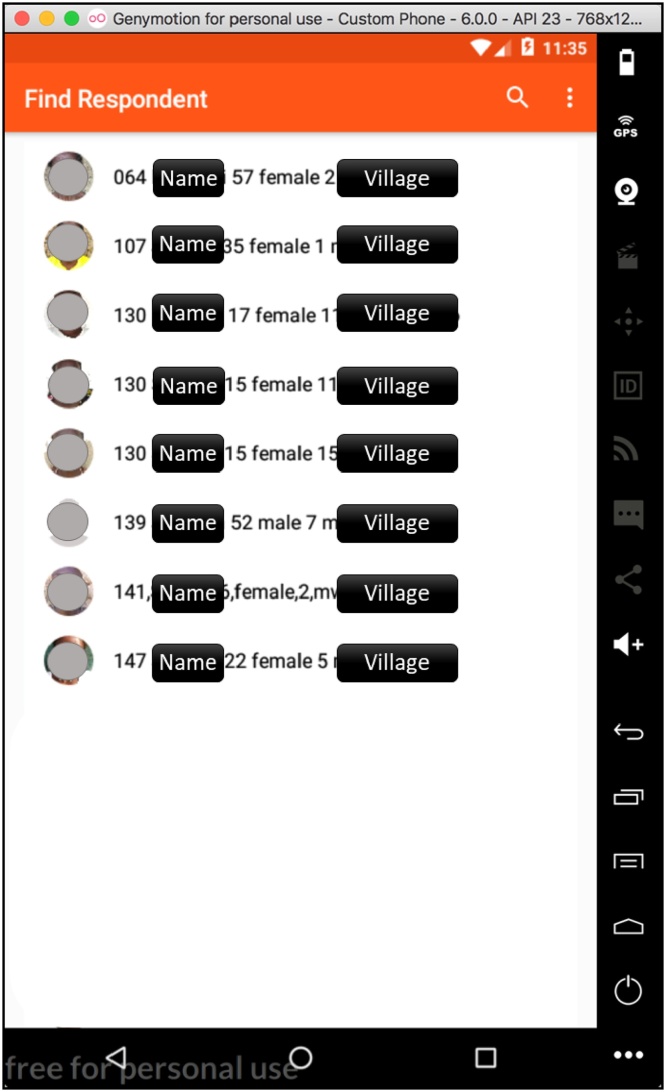
Trellis: Example of respondent search function.

One of the challenges in mapping social networks, especially in low literacy settings, is the difficulty of uniquely identifying individuals. To overcome this challenge, we created a photographic roster in each village and asked respondents to use the photos to disambiguate their alters. We used the *Trellis* software on Android smartphones to take pictures of people 15 years and older and assign each person a unique identifier. All photos were linked to respondents’ information on the roster, including name, age, gender, household area structure number (a collection of households), and household number. When the respondents named a person during the survey, they were asked to confirm the identity of the alter using the photo. This person’s identifier was entered in the survey along with his or her name. *Trellis*' respondent search function allowed for a person to be quickly located (see Fig. 5).

#### Wave 2: social network survey

A senior researcher at ICRHK served as the supervisor who oversaw the work of surveyors. The supervisor assigned a set of respondents to a surveyor, who then completed the assignment before being assigned a new set. The supervisor’s responsibility was to ensure the completion of the surveys assigned before assigning a new set of surveys or leaving the village. After each surveyor's device was fully in sync with the central server, the supervisor could check which respondents had been visited during the day of data collection.

#### Real-time monitoring

Our Kenyan research partners at International Center for Reproductive Health in Kenya were provided with a special report to do real-time monitoring. This report included a set of indicators to monitor, for each surveyor, the number of surveys assigned, the status towards completion of each survey, the number of surveys completed, date and time of the survey, duration, GPS location (latitude, longitude, altitude), and interview notes. Interview notes included information such as “married away”, “relocated”, “works and lives outside the EA [enumeration area], or “visited her mother who lives outside the EA till next month.”

Below are some of the Kenya research team’s reflections on the use of report:

**Table:** 

*Frequency of use*
• Daily
*How was it Helpful?*
• The spreadsheet provided a summary by aggregating and extracting values from the data collected. • It simplified the data into more manageable chunks of information that allowed us to see what we were doing right and where we needed to improve, e.g. the number of visits to a household and the outcome of these visits. • It helped to monitor the progress of data collection to ensure quality of data.
*What decisions did it help you make?*
• Identifying household which had not been reached or where repeat visits were needed. • Tracking the number of interviews completed by an enumerator on a daily basis. • We were able to account for all the household members in each village. The notes section was helpful in getting more information about incomplete interviews. Thus information was used to target households.
*Any proposed improvements?*
• It covered essential elements needed for tracking the metrics of the study.
*Any problems caused by the technology?*
• The technology worked well and no problem was encountered.

Given the multitude of reasons that may make a respondent unavailable, having access to individualized responder notes describing the status of the interview allows better control over the data collection process. In turn, better control can minimize the extent to which the researcher spends effort and time unproductively. In the next section we detail some of the real time monitoring activities performed by the Kenya research team.

##### Monitoring not-eligible individuals

As mentioned above, the *Trellis* app facilitates the monitoring of data collection process by allowing surveyors to add notes after each completed survey or at each attempt to complete a survey. This information allowed supervisors to keep track of the individual respondents in the roster and account for the situation where individuals initially included in the roster became incapacitated, died, or relocated in the time between the creation of the roster of respondents and data collection. Table 3 contains the information about the final dispositions in our survey according to AAPOR Standard Definition (American Association for Public Opinion Research, 2011).

**Table 3:** Data completion.

	Low MCPR	High MCPR	Total
**Interview**			
Complete	666	1303	1969
Partial	1	18	19
**Eligible, Non-Interview**			
Refusals	30	177	207
Respondent away/unavailable	177	227	404
Physically or mentally unable/incompetent	12	20	32
Other	25	69	94
**Not Eligible**			
Dead	6	9	15
Relocated	25	54	79
Duplicate records	31	27	58
Under age	3	10	13
**Total**	976	1914	2890

###### Course correction

Supervisors updated the individuals included in the roster and surveyors did not need to go back to collect data.

##### Monitoring eligible, non-interview individuals: refusals

Furthermore, close monitoring of the data collection process using *Trellis* provides the researcher with a host of descriptive information that can be used to expose issues in the data collection process. For example, Table 3 suggests that, while completion proportion is the same for low and high MCPR communities, a larger percentage of outright refusals (one of a number of reasons for non-interview) occurred in the high MCPR community. This can be further investigated to highlight either a community or a surveyor issue. An examination of Table 3 below shows a larger proportion of poor-quality data on the high MCPR side. Together, these observations point to the possibility of a surveyor issue (rather than a community one).

###### Course correction

To ensure the collection of high-quality data, supervisors provided a refresher training to surveyors, including advice such as: (1) Be respectful and aware that many women need their husbands’ approval to take the survey; (2) Illiterate participants can take the survey but need a literate witness to sign the consent form on their behalf; they will also need fingerprinting; (3) It is important to be tenacious – but not aggressive – when seeking to (re)schedule an interview with residents who are not at home (e.g., at work or school) or otherwise indisposed; (4) It is important to speak one-on-one with each participant (e.g. if a co-wife wanted to be a part of the interview, kindly inform her that she can be interviewed separately); and (5) It is important to refrain from conducting an interview with a respondent who shows symptoms of not being sober.

##### Monitoring eligible, non-interview individuals: respondent away/unavailable

**Fig. 6:**
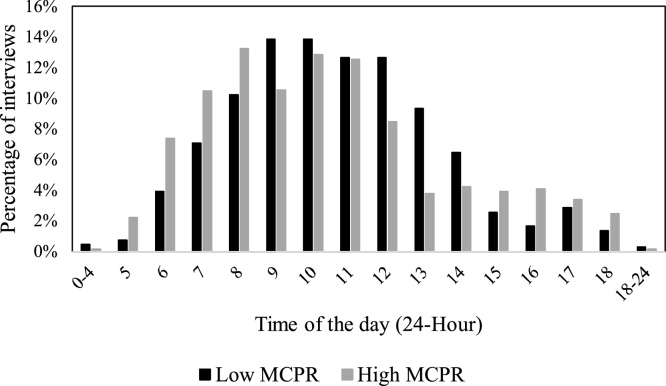
Respondents’ availability by time across two communities.

**Fig. 7:**
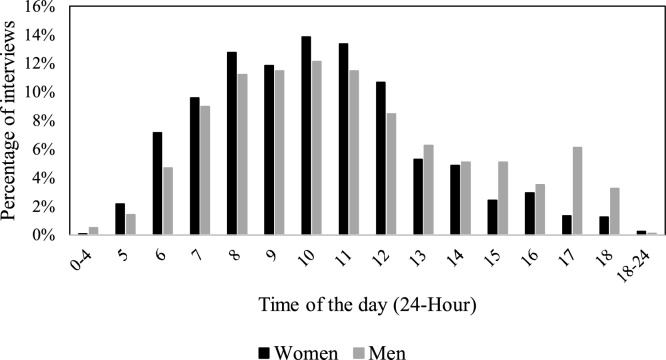
Respondents’ availability by gender.

There were cases were surveyors had to visit the same household multiple times in order to collect the data. For example, 14 % of the respondents couldn’t be “found again” during additional site visits. Of the 1,969 respondents who completed the survey, 14 % necessitated more than one visit (96 respondents were interviewed after two visits, 98 after 3 visits, 90 after more than three visits). The *Trellis* report also points out the times of the day that respondents can be recruited, which is particularly useful in contexts in which it is not clear what the schedule of the respondents will be. In the vast majority of cases, the interviews took place between 7 a.m. and 12 pm, with a peak between 8 a.m. and 11 a.m.. However, a closer look at the demographic data showed that respondents were typically available before 8 a.m. and after 3 pm in the high MCPR community, and between 9 a.m. and 2 p.m. in the low MCPR community. Respondents from both communities were available to the same degree between 10 am-12 pm. Female respondents were more available than men between 5 a.m. and 12 pm; men had greater availability after 1 pm, and particularly in the evenings. These observations are consistent with possible work patterns that can be different not only between communities but also between genders and seasons. Fig. 6, Fig. 7 provide a graphical illustration of respondents’ availability.

Additionally, almost all research that involves interviews faces the possibility that the respondent becomes unavailable. *Trellis* allowed for a close follow-up of these instances and provides the researcher with the necessary information to adjust his/her strategy and schedule (including, possibly, identifying contacts of the respondents who might be of help in locating them). For example, in our context, we learned that interviews had to be restarted 26 % of the cases. Most of the interviews restarted were completed in one day (25 %). However, in 1% (i.e., 13 cases), the interview was stopped and started days, even months later (from 2 up to 95 days’ delay). In 50 % of the restarted cases, the interview took place in a different location, but most locations were close to one another. In one of the most exceptional instances, the first part of the interview started at 11 a.m. in one location and the second part at 5 pm 2 km away.

###### Course correction

Supervisors instructed surveyors whether to go back to specific households, and based on the possible work patterns identified, supervisors instructed surveyors when to visit specific households. Finally, in 18 cases, supervisors assigned new surveyors to complete the interviews.

#### Assessing bias in social network data collection

The *Trellis* app also allowed us an unprecedented window to examine the data collection process by accounting for surveyor characteristics. This can be particularly useful when collecting data that deals with issues that can be considered sensitive or even taboo (the latter being our case). In our context, we benefited from the work of 11 surveyors in the field, out of which 8 surveyors were experienced, all had at least a college certificate, 4 were men and 7 were women, 6 were married and 5 were single. In general, male surveyors interviewed a similar number of men and women respondents while female surveyors interviewed more women than men. There were no differences in the number of respondents interviewed by married versus single surveyors.

**Fig. 8:**
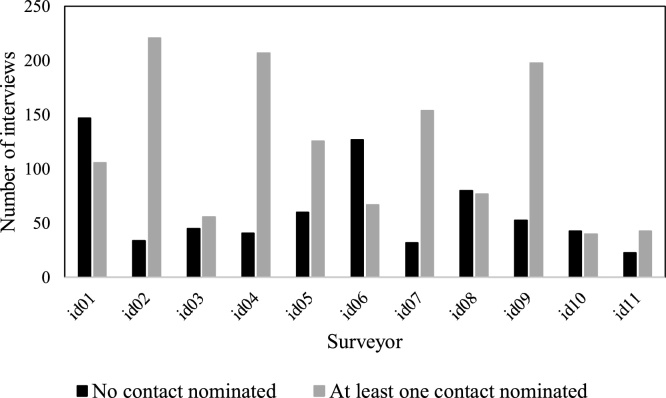
Number of interviews with contacts nominated for relation *Talk about MC*.

**Fig. 9:**
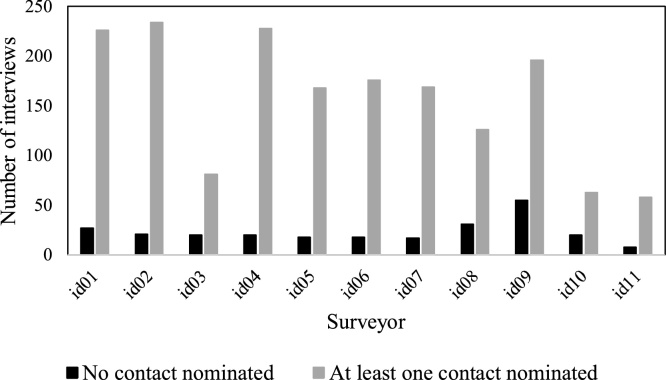
Number of interviews with contacts nominated for relation *Spend free time with*.

While examining the social network data collection, *Trellis* facilitated assessing differences at surveyor level in social network data collection based on the type of relation for which data were solicited. Fig. 8, Fig. 9 present the number of interviews where respondents provided at least one network contact for the relations *Talk about MC* and *Spend free time with* respectively. We can see that for surveyors *id01* and *id06* there are more respondents who did not provide contacts for *Talk about MC* relation than respondents who provided contacts. However, for relation *Spend free time with*, there are more respondents who provided contacts across all surveyors. Based on the observed difference in collecting network contacts between surveyor and question type, we conducted statistical tests to assess if these differences were systematically related to surveyor’s demographic characteristics. Therefore, we asked:

#### Are there any differences in collecting social network data based on surveyors’ social demographic characteristics?

##### Effect of surveyors’ experience

We used one-way ANOVA to detect statistically significant differences in the effect of surveyors’ demographic characteristics on the social networks data collection process. Results showed that experienced surveyors were more likely to elicit social network data for relations *Talk about MC* [F(1,1967) = 27.67, *p* < 0.001], *Talk about child spacing* [F(1, 1967) = 53.85, *p* < 0.001], *Seek health advice from* [F(1, 1967) = 57.48, *p* < 0.001], *Came for health advice* [F(1, 1967) = 57.58, *p* < 0.001], *Talk about private issues* [F(1, 1967) = 10.87, *p* < 0.01], *Borrow money from* [F(1, 1967) = 20.25, *p* < 0.001], and for the relation *Asked to borrow money* [F(1, 1967) = 10.66, *p* < 0.01]. For example, Table 4 shows that experienced surveyors collected information about MC network contacts in 69 % of the times, while medium experienced surveyors collected MC network data only in 57 % of the times. However, there was no difference in the effect of surveyor experience on eliciting responses for the social relation. Based on these results, we conclude that:

**Table 4:** One-way ANOVAs examining the impact of surveyor’s experience on social network collection.

Relation Type	Relation	Surveyor experience			
		Medium	Experienced	F-value	MS
MC and child spacing	Talk about MC	57 %	69 %	27.67***	6.15
	Talk about child spacing	55 %	72 %	53.85***	11.54
Health	Seek health advice from	66 %	82 %	57.48***	9.72
	Came for health advice	63 %	80 %	57.58***	10.42
Trust	Talk about private issues	74 %	82 %	10.87**	1.75
	Borrow money from	84 %	91 %	20.25***	1.87
	Asked to borrow money	83 %	89 %	10.66**	1.15
Social	Spend free time with	87 %	87 %	0.56	0.06

*Note.* Degrees of freedom for all analyses = 1, 1967; ∗∗∗p < 0.001; ∗∗p < 0.01; ∗p < 0.05.

*1. Experienced surveyors are more comfortable in collecting network data about taboo and private topics than less experienced surveyors.*

##### Effect of surveyors’ marital status

Second, one-way ANOVA suggests a statistical effect of surveyors’ marital status on collecting social network data. Married surveyors were more likely to elicit network data about taboo and private topics, such as *Talk about MC* [F(1, 1967) = 5.02, *p* < 0.05] and *Talk about child spacing* [F(1, 1967 = 8.65, *p* < 0.01]. However, those surveyors who were not married were more likely to collect data about *Spend free time with* [F(1, 1967) = 20.31, *p* < 0.001]. Table 5 presents the proportion of surveyors that collected network data based on their marital status. Based on these results, we conclude that:

**Table 5:** One-way ANOVAs examining the impact of surveyor’s marital status on social network collection.

Relation Type	Question	Surveyor marital status			
		Married	Single	F-value	MS
MC and child spacing	Talk about MC	68 %	63 %	5.02*	1.13
	Talk about child spacing	70 %	64 %	8.65**	1.89
Health	Seek health advice from	83 %	71 %	47.00***	7.99
	Came for health advice	80 %	70 %	31.08***	5.70
Trust	Talk about private issues	84 %	75 %	27.35***	4.36
	Borrow money from	90 %	88 %	0.09	0.26
	Asked to borrow money	87 %	88 %	0.001	0.00
Social	Spend free time with	83 %	91 %	20.31***	2.18

*Note.* Degrees of freedom for all analyses = 1, 1967; ∗∗∗p < 0.001; ∗∗p < 0.01; ∗p < 0.05.

*2a. Married surveyors are more comfortable in collecting network data about taboo and private topics than single surveyors.*

*2b: Single surveyors are more efficient in collecting network data about social relations than married surveyors.*

##### Effect of surveyors’ gender

Third, one-way ANOVA suggests a statistical effect of surveyors’ gender on eliciting social network data. In general, female surveyors were more likely to elicit social network data than males. However, there was no difference in the effect of surveyor gender on eliciting responses for social relations. Table 6 presents the proportion of surveyors that collected network data based on their gender. Based on these results, we conclude that:

**Table 6:** One-way ANOVAs examining the impact of surveyor’s gender on social network collection.

Relation Type	Question	Surveyor gender			
		Female	Male	F-value	MS
MC and child spacing	Talk about MC	68 %	59 %	13.59***	3.04
	Talk about child spacing	70 %	59 %	24.05***	5.23
Health	Seek health advice from	84 %	61 %	146.08***	23.66
	Came for health advice	82 %	58 %	135.60***	23.64
Trust	Talk about private issues	80 %	77 %	2.59	1.31
	Borrow money from	91 %	85 %	14.18***	1.31
	Asked to borrow money	89 %	82 %	20.26***	2.19
Social	Spend free time with	87 %	88 %	1.54	1.67

*Note.* Degrees of freedom for all analyses = 1, 1967; ∗∗∗p < 0.001; ∗∗p < 0.01; ∗p < 0.05.

*3: Female surveyors are more comfortable in collecting network data about taboo and private topics than male surveyors.*

##### Effect of surveyor-respondent gender homophily

Furthermore, given that women and girls in Kenya are exposed to cultural norms to have many children and given the stigma related to their use of modern contraceptive, we were interested in investigating whether female respondents were less or more open to provide information about their social network contacts on MC and child spacing relations. Results of one-way ANOVA show that female respondents are more likely to provide network contacts with whom they talk about MC when they are interviewed by female surveyors than when they were interviewed by male surveyors. Table 7 shows that 71 % of the female respondents that were interviewed by female surveyors nominated at least one contact. By contrast, only 62 % of the female respondents that were interviewed by male surveyors nominated at least one contact [F(1, 1967) = 10.21, *p* < 0.01]. There is no significant effect for the relation *Talk about child spacing.* Based on these results, we conclude that:

**Table 7:** One-way ANOVAs examining the impact of surveyor and respondent’s gender on social network collection.

Surveyor Gender	Relation	Respondent Gender			
		Woman	Man	F-value	MS
Woman^a^	*Talk about MC*	71 %	63 %	10.21**	2.19
	*Talk about child spacing*	72 %	69 %	1.69	0.35
Man^b^	*Talk about MC*	62 %	57 %	1.4	0.34
	*Talk about child spacing*	61 %	58 %	0.78	0.19

*Note.* ∗∗∗p < 0.001; ∗∗p < 0.01; ∗p < 0.05; ^a^ N = 1,968; Degrees of freedom = 1, 1367; ^b^ N = 600; Degrees of freedom = 1, 598.

*4: Female respondents are more comfortable in providing network data about MC to female surveyors than to male surveyors.*

##### Effect of surveyor-respondent gender homophily in low vs high MCPR community

**Fig. 10:**
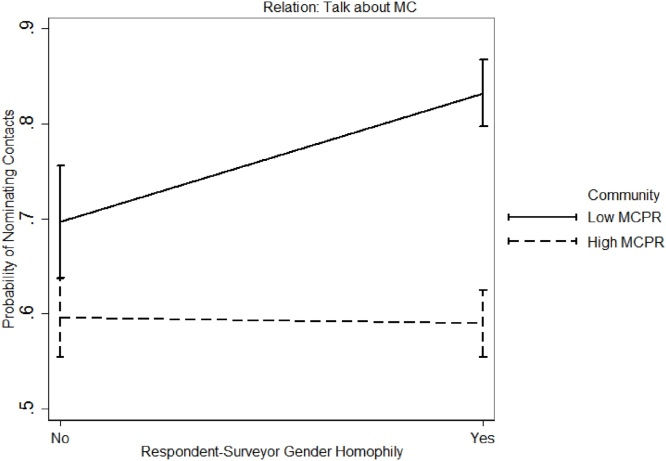
Interaction effect gender homophily and community for network relation *Talk about MC*.

**Fig. 11:**
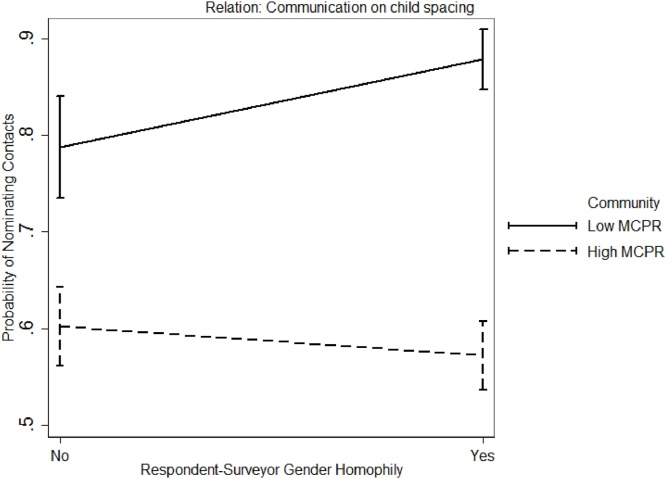
Interaction effect gender homophily and community for network relation *Communication on child spacing*.

Finally, we performed a two-way ANOVA to examine the effect of surveyor-respondent gender homophily in the two communities. Results suggest that the issue of surveyor-respondent homophily was more salient in the low MCPR community when collecting social network data about *Talk about MC* [F(2,1965) = 9.45, *p* < 0.01], and *Talk about child spacing* [F(2,1965) = 7.25, *p* < 0.01]. Fig. 10, Fig. 11 present the effect of respondent-surveyor gender on social network data collection by community. Based on these results, we conclude that:

*5: Female respondents are more comfortable in providing network data about MC to female surveyors than to male surveyors.*

We realize we are performing multiple (descriptive) analyses here and are thus simply reporting our observations, without adjustment for multiple comparisons. Future research on these issues, facilitated by the use of *Trellis*, should further investigate network data collection biases related to surveyor characteristics.

### Lessons learned

At the end of the data collection, the research team convened a group of family planning experts from Kenyan NGOs, Universities, County Departments of Health, and International Non-Governmental Organizations to share the study’s aims and methods, preliminary findings from qualitative and quantitative analyses, as well as computational modeling, and to seek input in interpreting our findings, how to make them actionable for health care policy and decision makers in Kenya. The meeting included a series of presentations by team members followed by immediate, facilitated discussions and participant reflection. Attendees demonstrated interest in the approaches used, actively engaged in the convening activities, and provided valuable feedback in terms of potential applications and interventions.

From the perspective of the potential applications of social network analysis, the main takeaways from the convening included: (1) confirmation of the assumption that social network analysis was a novel but much valued approach for Kenyan implementers and (2) the realization that participants had limited exposure to multi-level processes of change in general, and to social network interventions, in particular. We also took stock of the complexity of collecting whole-network data for the PMA2020 team –an experience that has been reported by others involved in sociometric data collection.

We derived several key benefits from collecting social network data using *Trellis*. *Trellis* enabled access to hard-to-reach populations. *Trellis* allowed surveyors to collect data in a local language (Swahili) and from low-literacy populations. It also allowed surveyors to disambiguate the identity of their respondents’ network alters by showing them their photographs. The surveyors were also able to record and track information about multiple (repeat) visits and split-interviews with participants into multiple sessions. *Trellis* offered real-time monitoring of data collection efforts via a dashboard to assess the performance of the surveyor staff, tracking the location and time spent by the surveyors, as well as noting any variability or biases between surveyors (such as differences in survey administration processes). This enabled agile adjustments to survey administration. Using *Trellis* provided valuable additional information for researchers to better report data collection processes and to identify limitations in the datasets they gathered. These included detailed records on repeat and/or truncated visits, interview duration (for various segments of the survey) and variability in these measures across surveyors, time of day, and specific geographical location. Finally, the time-stamp associated with each respondent’s recall of their social network alters enabled us to ask challenging research questions about the cognitive processes invoked by individuals and to explain the sequence and patterns by which individuals reconstruct their network.

## Conclusion

We have sought to demonstrate the efficacy of a mobile social networking platform, *Trellis*, to address the challenges associated with collecting large-scale sociocentric social network data from populations that vary in literacy, especially in hard-to-reach and underserved sectors of society. Specifically, we described the application of *Trellis* to map two rural villages in Kenya, conduct a photographic census of the population, and collect health and social network data from all 1,969 respondents over age 15 in these villages. We demonstrated how *Trellis* facilitates innovative research by offering four key benefits. *First*, *Trellis* enables access to hard-to-study populations allowed surveyors to collect data in multiple languages (Spanish, Swahili, English) from a population with low literacy. It allowed surveyors to disambiguate the identity of their respondents’ network alters by showing them their photographs. The surveyors were also able to record and track information about multiple (repeat) visits and facilitate splitting interviews with participants into multiple sessions.

*Second*, *Trellis* offers real-time monitoring of data collection efforts via a report to assess the performance of the surveyor staff, as well as noting any variability or biases between surveyors (such as differences in survey administration processes). This enabled agile adjustments to survey administration.

*Third, Trellis* enables collection of data in locations with limited access to wireless networks and is fully functional even when offline. The data collection process is followed by the synchronization with the central server when the device is returned to an area with network connectivity.

*Finally*, using *Trellis* provides valuable additional information for researchers to better report data collection processes and to identify limitations in the datasets they have gathered. These include records of the time stamp associated with each respondent’s recall of their social network alters also enables researchers to ask heretofore challenging research questions about the cognitive processes invoked by individuals to explain the sequence and patterns by which individuals reconstruct their network.

In the case described here, the challenge was to be able to unleash the potential of using social network theories and methods to explain attitudes and behaviors about family planning in rural communities in Kenya. However, the design features provided by *Trellis* can be easily generalizable to a wide variety of contexts and communities that include, but are not restricted to, hard-to-reach populations (*Trellis* has also been used in a poor, mountainous region in western Honduras, for instance Shakya et al., 2017b). Our use of *Trellis* benefited from collecting *a priori* a complete census list of members in the overall network. However, *Trellis* also has the option for respondents to add the names (or pseudo-names) of alters not on the roster and add information about these alters. While this feature was less frequently used in the present study it might be particularly helpful if *Trellis* were to be used to collect ego-centric network data from other hard to reach populations such as homeless, sex workers and injection drug users, not already on the roster. While there have been promising advances in using web-based data collection approaches (Stark and Krosnick, 2017), *Trellis* builds on these efforts by providing a mobile platform to assist surveyors (rather than directly for respondents), being sociocentric (rather than egocentric), easy to implement in multiple languages by administrators of surveys, easy to learn how to use by the surveyors themselves, easy to serve as a report monitor for those managing the survey, and flexible to use in low tech environments without persistent Internet conductivity. The high-resolution metadata (data about the data collection process itself) provides an unprecedented opportunity to easily evaluate and include in studies the strengths and limitations of the data collection process. The metadata also offers the opportunity for researchers interested in the measurement of social network data to understand possible sources of error, as well as lack of reliability and validity in the data collected.

The past decade has witnessed considerable excitement about the possibility of using computational social science that leverages digital trace data to understand social processes occurring in the 21 st century (Contractor, 2019; Lazer et al., 2009). However, many key insights generated from social network data are still based on data collected from social network surveys. It remains an open question if we can use digital trace data to infer what individuals would say when responding to on a social network survey that also contains many more variables about the lives of the subjects (Leonardi and Contractor, 2018). These challenges are especially important in communities that are hard to reach. We must continue to leverage technologies to find more effective ways of collecting face-to-face social network data. *Trellis*, which is publicly available, offers significant opportunities along this trajectory.

## Footnotes

1 http://trellis.yale.edu/

2 www.openstreetmap.org

3 https://tools.ietf.org/html/rfc4122

4 This function is available starting with Trellis version 3.

5 https://en.wikipedia.org/wiki/JSON

6 https://www.gatesfoundation.org/what-we-do/global-development/family-planning
